# Accuracy of functional near-infrared diffuse optical spectroscopy to aid in the differentiation of depressive symptoms

**DOI:** 10.3389/fpsyt.2025.1496675

**Published:** 2025-08-08

**Authors:** Masaru Nakamura, Takahiko Nagamine

**Affiliations:** ^1^ Department of Psychiatric Internal Medicine, Kosekai-Kusatsu Hospital, Hiroshima, Japan; ^2^ Department of Psychiatric Internal Medicine, Sunlight Brain Research Center, Yamaguchi, Japan

**Keywords:** functional near-infrared spectroscopy (fNIRS), depression, auxiliary psychiatric diagnosis, state marker, trait marker

Mental health disorders represent a growing public health concern, yet their clinical evaluation frequently relies on subjective questionnaires. Functional near-infrared spectroscopy (fNIRS) offers a more quantitative approach by measuring cerebral hemodynamics (e.g., blood oxygenation, blood flow, etc.), which are linked to brain function and psychiatric symptoms. The present editorial, published in this journal (continued in Volume 1), focuses on fNIRS methods for the assessment of psychiatric disorders and brain function, and for the diagnosis of psychiatric disorders. It covers and presents research from November 2022 to May 2024 among the most cited papers, making it a very valuable paper for considering the potential of fNIRS (1). The efficacy of fNIRS in identifying disease-dependent alterations in cerebral blood flow within neural regions, as well as variations in functional connections between neurons, in both infants and adults, has been well-documented (1).

However, our clinical experience suggests that the diagnostic accuracy of auxiliary diagnoses of psychiatric disorders using fNIRS is lower than the reported values. The concordance rate between fNIRS diagnoses and psychiatric diagnoses was 44.0% for bipolar disorder and 38.2% for major depressive disorder, with approximately half of the cases classified as different disorders by fNIRS (2). This study was approved by the Ethics Committee of the Sunlight Brain Research Center, approval number SBRC-505. The influence of factors such as blood electrolytes and the dosage of antidepressants on the fNIRS waveform is also a subject of investigation (**Supplementary Table 1**).

A substantial body of research has asserted the efficacy of fNIRS in differentiating psychiatric disorders by employing a comparison with healthy individuals as a basis for this assertion (3). While fNIRS shows promise in identifying differences between healthy individuals and those with psychiatric disorders, using it to distinguish between specific disorders like schizophrenia, bipolar disorder, and major depressive disorder (especially when all present with depression) is a much more complex problem. The following are possible reasons why fNIRS has a low diagnostic rate as an auxiliary diagnostic for psychiatric disorders. First, the presence of shared symptoms, particularly depression, among these disorders complicates the identification of distinct neural signatures that are unique to each disorder. Second, the brain activity patterns associated with depression itself might mask the more subtle differences between the underlying disorders. Third, each of these disorders is itself quite heterogeneous, with the potential for subtypes within each category and individuals experiencing varying levels of severity. This heterogeneity poses a significant challenge in identifying consistent biomarkers for each disorder, as it hinders the ability to differentiate between the neural signatures of the disorder and the effects of medication. The tasks employed during fNIRS assessments can influence brain activation patterns, further complicating the analysis. The selection and standardization of tasks is paramount for effective comparison of results across individuals and studies. Performance on cognitive tasks is influenced by factors such as motivation, attention, and effort, which can introduce variability and compromise the reliability of results. While fNIRS demonstrates adequate temporal resolution, capable of tracking changes in brain activity over time, its spatial resolution is constrained. This limitation can impede the ability to identify and localize specific brain regions implicated in various disorders, thereby complicating the differentiation between them. The analysis of fNIRS data to differentiate between these disorders necessitates the employment of sophisticated statistical and machine learning techniques. The identification of the most pertinent features in the data that can reliably distinguish between the disorders can be a challenging endeavor. Consequently, the present approach of utilizing fNIRS for auxiliary psychiatric diagnosis can be effectively employed as a state marker rather than a trait marker for mental illnesses that indicate depression. Our study has several limitations, one of which is that it is a retrospective study. Psychiatrists’ clinical diagnoses were made in semi-structured interviews during routine medical practice, not for research purposes. The Structured Clinical Interview for DSM-5, Research Version (SCID-5-RV) was not used. As a result, there may be a small number of cases in which bipolar depression was misdiagnosed as unipolar depression, and there may be cases in which the NIRS diagnosis and clinical diagnosis were consistent but were not. Second, our study did not use a scale that examines the degree of depression, such as the Hamilton Depression Rating Scale (HAM-D). It has been shown that the accuracy of NIRS increases when the HAM-D score is 8 points or higher. However, our study patients were hospitalized with a GAF score of around 40, and as this value is often close to a HAM-D score of 20, their depressive state was likely suitable for differentiation using NIRS. Despite these limitations, psychiatric diagnosis using NIRS is underdeveloped and research is still needed to improve its accuracy for use in adjunctive psychiatric diagnosis.

In conclusion, while fNIRS is a valuable tool for studying brain function in psychiatric disorders, using it to differentiate between similar disorders, such as schizophrenia, bipolar disorder, and major depressive disorder, is a significant challenge. Further research is necessary to develop more effective tasks, improve data analysis techniques, and account for the factors that can contribute to variability in brain activity. A combination of fNIRS with other neuroimaging techniques, such as electroencephalography or magnetic resonance imaging, may also be necessary to improve diagnostic accuracy, as demonstrated in the editorial by Shang et al. fNIRS is a relatively inexpensive tool with a wide range of applications in daily psychiatric clinical practice. However, further research is necessary to ascertain its role as an auxiliary diagnostic tool for psychiatric disorders.
